# Dissecting Tumor-Immune Microenvironment in Breast Cancer at a Spatial and Multiplex Resolution

**DOI:** 10.3390/cancers14081999

**Published:** 2022-04-14

**Authors:** Evangelos Tzoras, Ioannis Zerdes, Nikos Tsiknakis, Georgios C. Manikis, Artur Mezheyeuski, Jonas Bergh, Alexios Matikas, Theodoros Foukakis

**Affiliations:** 1 Department of Oncology-Pathology, Karolinska Institutet, 171 77 Stockholm, Sweden; ioannis.zerdes@ki.se (I.Z.); nikolaos.tsiknakis@ki.se (N.T.); gmanikis@gmail.com (G.C.M.); jonas.bergh@ki.se (J.B.); alexios.matikas@ki.se (A.M.); 2 Breast Center, Theme Cancer, Karolinska University Hospital, 171 77 Stockholm, Sweden; 3 Computational Biomedicine Laboratory, Institute of Computer Science, Foundation for Research and Technology Hellas (FORTH), 700 13 Heraklion, Greece; 4 Department of Immunology, Genetics and Pathology, Uppsala University, P.O. Box 516, 751 20 Uppsala, Sweden; artur.mezheyeuski@igp.uu.se

**Keywords:** immune microenvironment, breast cancer, heterogeneity, longitudinal, multiplex, spectral imaging, artificial intelligence

## Abstract

**Simple Summary:**

The evaluation of breast cancer immune microenvironment has been increasingly used in clinical practice, either by counting tumor infiltrating lymphocytes or assessing programmed death ligand 1 expression. However, the spatiotemporal organization of anti-breast cancer immune response has yet to be fully explored. Multiplex in situ methods with spectral imaging have emerged to deconvolute the different elements of tumor immune microenvironment. In this narrative review, we provide an overview of the impact that those methods have, to characterize spatiotemporal heterogeneity of breast cancer microenvironment at neoadjuvant, adjuvant and metastatic setting. Multiplexing in situ can then be useful for new classifications of tumor microenvironment and discovery of immune-related biomarkers within their spatial niche.

**Abstract:**

The tumor immune microenvironment (TIME) is an important player in breast cancer pathophysiology. Surrogates for antitumor immune response have been explored as predictive biomarkers to immunotherapy, though with several limitations. Immunohistochemistry for programmed death ligand 1 suffers from analytical problems, immune signatures are devoid of spatial information and histopathological evaluation of tumor infiltrating lymphocytes exhibits interobserver variability. Towards improved understanding of the complex interactions in TIME, several emerging multiplex in situ methods are being developed and gaining much attention for protein detection. They enable the simultaneous evaluation of multiple targets in situ, detection of cell densities/subpopulations as well as estimations of functional states of immune infiltrate. Furthermore, they can characterize spatial organization of TIME—by cell-to-cell interaction analyses and the evaluation of distribution within different regions of interest and tissue compartments—while digital imaging and image analysis software allow for reproducibility of the various assays. In this review, we aim to provide an overview of the different multiplex in situ methods used in cancer research with special focus on breast cancer TIME at the neoadjuvant, adjuvant and metastatic setting. Spatial heterogeneity of TIME and importance of longitudinal evaluation of TIME changes under the pressure of therapy and metastatic progression are also addressed.

## 1. Introduction

The evasion of immune response is considered a hallmark of cancer and efforts have focused on re-directing immune reaction against malignant cells [1]. The field of cancer immunotherapy has been revolutionized by the introduction of immune checkpoint inhibitors (ICI) that have been successfully used for the treatment of various malignant neoplasms [2,3,4,5]. However, not all patients benefit and ICI are associated with immune-related adverse effects, which can sometimes be severe [6]. Hence, predictive biomarkers are needed to identify potential responders and avoid unnecessary toxicities. In breast cancer (BC), studies have mostly focused on triple negative (TNBC) disease both in metastatic [7,8,9] and early-stage settings [10,11]. Predictive biomarkers are still lacking as PD-L1 positivity did not predict benefit from ICI in the neoadjuvant setting [10,11] and produced discordant results in metastatic BC patients [7,8].

Nonetheless, anti-tumor immune response is an important player in BC pathophysiology [12,13,14]. In clinical settings, anti-cancer immunity has been mostly assessed by hematoxylin and eosin (H&E) evaluation of tumor infiltrating lymphocytes (TILs) and immunohistochemical (IHC) evaluation of programmed death ligand 1 (PD-L1). For example, in a pooled analysis of 3771 early BC patients, increased rates of TILs predicted response to neoadjuvant chemotherapy and was associated with improved survival in HER2-positive BC (HER2+ BC) and TNBC [15]. In addition, immune-related gene expression profiles have proven to be prognostic in BC in both early and metastatic settings [13,14]. However, all the aforementioned immune-related biomarkers are just surrogates of an underlying tumor inflammation [16,17]. In addition, single biomarker expression demonstrated only modest performance, because of analytical limitations and tumor immune microenvironment (TIME) biology. In the case of the widely studied biomarker, PD-L1, assessment exhibits great inter-observer variability, given the different antibodies, platforms and scoring systems that exist [18]. Besides this, PD-L1 is but one immune checkpoint and interactions within TIME are way more complex [19]. Furthermore, immune signatures are devoid of spatial information and the generated signal is dominated by the most abundant cellular population [20], while hematoxylin and eosin (H&E) evaluation of TILs is a bulk measurement of stromal lymphocytic infiltration (sTILs). Although guidelines have been established [21,22], some level of interobserver variability still exists [23]. Intriguingly, spatial heterogeneity of TILs is an important factor affecting the interobserver variability [24]. Thus, better understanding of TIME organization and function—which remains limited—is considered essential for identifying patients with a pre-existing anti-tumor immune response [25].

In order the aforementioned challenges to be overcome, evaluation of subpopulations [26,27], functional status [28] and spatial heterogeneity [29] of immune infiltrate should be taken into consideration. Furthermore, tumor evolution over the course of treatment or progression to metastatic disease contributes to the complexity by introducing temporal heterogeneity both in the tumor and TIME [30]. Thus, a promising approach for assessing TIME is to combine multiple biomarkers evaluation taking into account the spatial and temporal context [25]. This is supported by a large meta-analysis that included 8135 patients of more than ten different solid tumors, treated with ICI. It was demonstrated that fluorescent multiplex immunohistochemistry (mIHC/IF) alone, outperforms PDL1 immunohistochemistry, immune signatures and tumor mutational burden in terms of sensitivity and positive predictive value for prediction of response to ICI [31]. Towards this end, novel multispectral and spatial technologies should be used. Several multiplex in situ methods have been recently developed and gaining attention, allowing for the in situ detection of multiple targets within spatial context [20]. In this review, we aim to provide an overview of different multiplex in situ assays applied in breast cancer research. Spatial characterization of breast cancer TIME is also discussed, specifically in relation to longitudinal evaluation under treatment pressure as well as from primary to metastatic tumors.

## 2. Multiplex In Situ Methods: A Plethora of Assays Platforms and Analysis Tools

Recent advances in spatial technologies and emerging multiplex approaches have transformed the landscape of quantitative in situ profiling in cancer, from single to multiple biomarker assessment. The various protein-based multiplex in situ methods are summarized in Table 1, while an overview, description of their capacity and suitable software is provided hereunder in detail.

### 2.1. Antibody Conjugated with Chromogen

Multiplex chromogenic immunohistochemistry (mIHC) is an evolutionary form of conventional chromogenic IHC that allows the detection of different proteins at the same tissue section. Its main advantages include the pathological assessment by using conventional brightfield microscopy, whole slide visualization and cost-effectiveness, since no special equipment is needed. Such approaches are very likely to enter clinical practice in the immediate future. However, utility can be held back by image analysis limitations due to chromogens nature, cross-reactivity between primary antibodies from different staining solutions, slow scanning capacity, visual sight limitations and semi-quantitative nature of IHC. To date, the two most commonly used mIHC methods coupled with chromogen are Discovery Ultra [32] (Roche Diagnostics, Rotkreuz, Switzerland) and Multiplexed Immunohistochemical Consecutive Staining on Single Slide (MICSSS) [33].

#### 2.1.1. Discovery Ultra

Discovery Ultra is a commercially available mIHC platform for formalin-fixed paraffin-embedded (FFPE) sections [32]. Antibody staining is automated and sequential in a way that a new antibody is used without removal of the previous one. With this platform, up to five protein markers can be detected by using new-generation chromogenic dyes. In brief, a classic chromogen 3,3′-diaminobenzidine (DAB) is utilized, which is a scatterer of light, not an absorber, and has a very broad spectrum, with characteristics that change depending of staining intensity [34]. Combination of three such chromogens in one slide can fill the human-eye visible spectrum; hence, new chromogens can produce staining patterns with more narrow absorbance spectrum to be distinguished easier. The main disadvantage of this method is the limited number of co-localized biomarkers, two or three, that can be visually assessed.

#### 2.1.2. Multiplexed Immunohistochemical Consecutive Staining on Single Slide (MICSSS)

Multiplexed Immunohistochemical Consecutive Staining on Single Slide (MICSSS) represents another mIHC method that uses sequential cycles of IHC staining, coverslip mounting, image scanning, coverslip removal and de-staining performed on a single slide [33]. A whole-slide scanner is used for scanning after each staining. Up to ten markers can be visualized with brightfield microscopy and a multiplex image is created by aligning together individual digitalized IHC images. The main disadvantages of this methodology are the time-consuming protocol, the inability to assess biomarker intensity, the possibility of cross-reaction of antibodies and the limitations accompanying chromogenic dyes. Furthermore, the procedure is prone to human error as staining is not automated and careless coverslip removal can result in tissue damage.

### 2.2. Antibody Conjugated with Fluorophore

Multiplex immunofluorescence immunohistochemistry (mIF/IHC) comprises methods that use antibodies conjugated with fluorophores [35,36,37]. Fluorophores have narrow emission and absorbance spectra compared with chromogens and allow distinction of several proteins in situ [38]. These methods enable protein quantification and immunophenotypic interrogation even at subcellular level. Vectra^®^ Polaris™ (Akoya Biosciences, Marlborough, MA, USA) is a commercially available, automated, multiplex digital pathology tool that detects antibodies conjugated with fluorophores [35]. The detection is highly specific and can capture the expression of up to nine biomarkers at the same protein panel. Tissue FFPE slides can be stained in a fully automatic way. In brief, a specific primary antibody recognizes its distinct epitope; a secondary antibody is conjugated with polymers of horseradish peroxidase (HRP), enabling signal amplification; tyramides, conjugated to fluorophores, are converted by HRP into highly reactive oxidized intermediates, which bind covalently to tyrosine residues in the underlying tissue in close proximity to the epitope; then, primary and secondary antibodies are stripped away and the cycle repeats for the next target, while the covalently-binded fluorophores remain intact. The spectral imaging allows for the collection of detailed spectral characteristics of each scanned pixel. The captured images are processed by the compatible software inForm^®^ (Akoya Biosciences), using reference library of the emission spectrums of the individual fluorophores and autofluorescence, thus, unmixing the original multi-layer images into several channels. Samples remain undisrupted and can be reused for future studies. This technology provides spatial analysis and quantification of phenotypes in situ in a single slide. Vectra Polaris can achieve spatial resolution at level as low as 0.25 μm, making single cell resolution possible. The main advantages are the fully automated staining and scanning, and image analysis by the InForm software. Opal (Roche Tissue Diagnostics, Oro Valley, AZ, USA and Akoya Biosciences, Marlborough, MA, USA) is another iterative, mIF/IHC method [39]. The primary antibodies are conjugated with Opal fluorophores equipped with tyramide molecules. In this way, low abundance epitopes are detected, while the risk of antibody cross-reactivity decreases. Image acquisition is mediated with Vectra Polaris, multispectral unmixing with inForm, and quantitative analysis with QuPath software [40].

**Table 1:** Differences and comparisons of the various characteristics among the different multiplex platforms and assays.

Assay	D.Ultra [32]	MICSSS [33]	Vectra/Polaris [35]	Opal [39]	CODEX [41,42]	InSituPlex [43]	GeoMx DSP [44,45]	MIBI-TOF [46,47]	Cy-TOF [48]
**Vendor**	Roche	Remark et al.	Akoya Biosciences	Roche and Akoya Biosciences	Akoya Biosciences	Ultivue	Nanostring	IonPath	Bodenmiller et al.
**Antibody** **Conjugate**	chromogen	chromogen	fluorescent	Opal fluorescent	DNA barcode	DNA barcode	DNA barcode	Metal isotope	Metal isotope
**Tissue** **disruption**	no	no	no	no	no	no	no	Yes (adjustable)	yes
**Biomarkers**	5	10+	∼9	∼8	40+	∼5	70+	30+	30+
**Staining** **cycles**	Iterative	Iterative	Iterative	Iterative	Iterative	1	1	1	1
**Scanning**	camera	camera	Vectra Polaris	Vectra Polaris	CODEX fluidics	compatible with various systems	GeoMx	TOF spectrometer	TOF spectrometer
**Profiling** **area**	Whole slide	Whole slide	adjustable (660 μm${}^{2}$ → whole slide)	adjustable (660 μm${}^{2}$ → whole slide)	660 μm${}^{2}$	NA	adjustable (280 μm${}^{2}$ → 800 μm${}^{2}$)	800 μm${}^{2}$	1 mm${}^{2}$
**Software**	NA	NA	InForm	InForm QuPath	CODEX analysis manager & viewer	compatible with various systems	GeoMx data analysis suite	MIBIAnalysis	HistoCat, Cell Profiler
**Resolution**	Human vision	Human vision	0.25–0.9 μm	0.25–0.9 μm	0.26 μm	NA	10 μm	0.26 μm	1 μm
**Spatial** **information**	yes	yes	yes	yes	yes	yes	limited	yes	yes
**Single-cell** **information**	yes	yes	yes	yes	yes	yes	limited	yes	yes
**Disadvantages**	Human Vision Limits, Semi- Quantitative	time- consuming, coverslip removal errors	costly, limited marker numbers	costly, limited marker numbers	costly, time- consuming, difficult cell segmentation, difficult panel validation	limited marker numbers	costly, limited spatial information, limited single cell information	special training, costly, time- consuming, difficult cell segmentation, difficult panel validation	special training, costly, time- consuming, difficult cell segmentation, difficult panel validation

Although fluorophores are generally considered more versatile than chromogens, their use suffers from three main issues: signal “spectral bleed”, FFPE intrinsic autofluorescence and tyramide-based chemistry limitations [38]. Firstly, hundreds of fluorophores are commercially available but signal “bleed-through” artifact and spectral overlap can occur if fluorophores with too similar emission wavelengths are used together, resulting in false positive staining [49]. Secondly, FFPE intrinsic autofluorescence should be taken into consideration prior to fluorophore selection, as it can lower the sensitivity of fluorescent staining. Intrinsic autofluorescence is mostly developed during formalin fixation of FFPE and has peak emission spectrum in the 475–500 nm range [50]. Several approaches can be used to overcome FFPE autofluorescence, including autofluorescence quenching by chemical or physical means, avoiding emission spectra near the autofluorescence peaks and use of multispectral imaging with spectral signal unmixing [51]. Thirdly, methods that use tyramide based chemistry for signal amplification can suffer from the so-called “umbrella effect”: binding of tyramide reagents to epitopes can potentially interfere with the binding of a sequential primary antibody, leading to false-negative results. This is particularly evident when multiple proteins are located in the same subcellular compartment and can limit the number of evaluable biomarkers [49]. Overall, mIF/IHC techniques have the potential to become useful in routine diagnostics. However, before clinical use, standardization and validation of staining and image analysis protocols are essential.

### 2.3. Antibody Conjugated with DNA-Barcode

#### 2.3.1. CO-Detection by indEXing (CODEX^®^) & InSituPlex^®^

CODEX^®^ (Akoya Biosciences, Marlborough, MA, USA) is a commercially available method that uses DNA conjugated antibodies [41,42]. CODEX detects antibodies labeled with unique DNA-barcodes. The detection is highly specific and can simultaneously capture the expression of more than 40 biomarkers. Tissue FFPE slides are stained in a single step with the antibody panel. Consecutive cycles of labeling, imaging and removing barcodes are performed in a fully automatic way by the CODEX fluidics instrument. The collected images are then integrated into one, and image analysis is performed by CODEX analysis manager and CODEX Analysis Viewer software. Tissue samples remain undisrupted and can be reused for future studies. This technique provides the opportunity for simultaneous assessment of expression and spatial distribution of multiple biomarkers in situ on a single slide. CODEX can achieve spatial resolution at a level as low as 0.26 μm, making single cell resolution possible. It has the advantage of being compatible with existing fluorescent microscopes for image generation. Although this platform can achieve comprehensive high-level multiplexing, its use is held back by its high cost, limited throughput and restriction to only small region of interest (ROI) on the slide available for the imaging. InSituPlex^®^ (Ultivue, Cambridge, MA, USA) is another multiplex in situ method that uses antibodies conjugated with DNA-barcodes [43]. Tissue FFPE slides are incubated with the primary antibody mixture in one step. After barcode amplification, a mixture of complementary, fluorophore-linked probes hybridizes each barcode, augmenting the signal. The technique is compatible with various scanning and image analysis systems.

#### 2.3.2. GeoMx^®^ Digital Spatial Profiling

GeoMx^®^ Digital Spatial Profiling (DSP) (Nanostring, Seattle, WA, USA) is a commercially available method that uses DNA-barcode conjugated antibodies [44,45]. In this platform, two types of antibodies are used: compartment-defining antibodies conjugated with fluorophores to morphologically select ROIs, and antibodies conjugated with unique oligonucleotide barcodes for multiplexing. This high-throughput technique provides simultaneous assessment of expression of multiple biomarkers in situ and in a compartment-based manner within selected ROIs, determined by fluorescently conjugated antibodies. Tissue slides are stained in a single step with the antibody mastermix. Then, UV light cleaves the barcodes from the antibodies, and the barcodes are collected in a microplate and quantified by the commercially available NanoString nCounter system. After quantitative analysis, the pool of antibodies originate back to their corresponding region of interest to allow spatial characterization. The detection can capture the expression of more than seventy biomarkers at the protein level, despite considerable amount of non-specific antibody binding and limited number of validated antibodies, although the various unique Nanostring barcodes. DSP could be useful for evaluating the expression of multiple immune regulators, since the spatial heterogeneity is addressed by examining stromal and tumoral compartments, as well as multiple ROIs. However, DSP provides limited spatial characterization of cell immunophenotypes, due to narrow “morphology-panel” selection, consisting mainly by three or four markers, such as cytokeratin and CD45 [38]. Hence, within a ROI, the DSP method could potentially suffer from missing rare cellular subpopulations, as in bulk-omics methods. Heatmaps of biomarker expression can be used to “visualize” the ROI but such an approach is costly for a whole slide. Although this platform can achieve comprehensive high-level multiplexing, high cost and limited spatial information represent some of its drawbacks.

### 2.4. Antibody Conjugated with Metal Isotope: Mass Spectrometry Immunohistochemistry (MS-IHC)

#### Imaging Mass Cytometry with Time of Flight (Cy-TOF) and Multiplex Ion Beam Imaging with Time of Flight (MIBI-TOF)

Mass Spectrometry Immunohistochemistry (MS-IHC) is an umbrella term for the next-generation methods combining mass cytometry with IHC aiming to characterize multiplex cellular immunophenotypes in situ [38]. Two landmark methods have been recently described, with the ability to detect more than 30 proteins at single cell level: (i) Imaging Mass Cytometry with Time of Flight (Cy-TOF) [48] and (ii) Multiplex Ion Beam Imaging with Time of Flight (MIBI-TOF) [46,47]. In both approaches, the FFPE slide is stained once with a heavy metal-conjugated antibody mastermix. Energy beam mediated, pixel-by-pixel tissue ablation releases the metallic isotopes that are then quantified by a TOF mass spectrometer and mapped back to the corresponding ROI [52]. For every marker, each pixel of the generated digital image depicts protein abundance. Individual images need then to be merged together for multiplexed imaging. In theory, both methods can detect more than 100 proteins simultaneously, but face limitations due to the number of validated antibodies. Although quite similar, the two methods differ in terms of resolution and level of tissue-ablation. In Cy-TOF, after one scanning, tissue destruction is mediated by a UV laser beam which achieves resolution at 1 μm. In MIBI-TOF an ion beam—usually consisted of rasterizing oxygen duoplasmatron—is used which achieves resolution up to 0.26 μm. Hence, tissue destruction can be adjusted depending on the desired resolution and scanning can be performed additional times. Although this platform can achieve comprehensive high-level multiplexing, its use is held back by its high cost, long scanning time, restriction to only small region of interest (ROI) on the slide available for the imaging and requirement of specially trained personnel.

### 2.5. Image Analysis Software for Multiplex Profiling Assays

Apart from interobserver variability, visual evaluation of multiplex stainings is limited to few markers, especially when co-expressed in the same cellular compartment. Thus, the development and use of relevant software programs in order to facilitate the analysis of multiplex images is of utmost importance. From a technical perspective, this can be achieved by leveraging image analysis and artificial intelligence (AI) tools in multiplex studies, subsequently activating an extensive analysis pipeline comprising of the following steps: (a) multispectral image decomposition and the unmixing of the different spectral components (i.e., markers) into separate images, (b) morphological tissue segmentation into distinct compartments (e.g., tumor, stroma), (c) nuclei and cytoplasm identification followed by the cellular segmentation, (d) cell phenotyping across the different types, and (e) spatial analysis on cell images to investigate potential associations between the different cell types [53]. Nowadays, there is a growing interest to incorporate these tools into TIME contexture analysis studies. Although a thorough presentation of these tools is far beyond our scope here and has been previously reviewed elsewhere [37,54,55]. Table 2 summarizes some of the most well-known and commercially available software packages [37,40,56,57,58,59] that embed AI technology into their analysis workflow.

**Figure 1:**
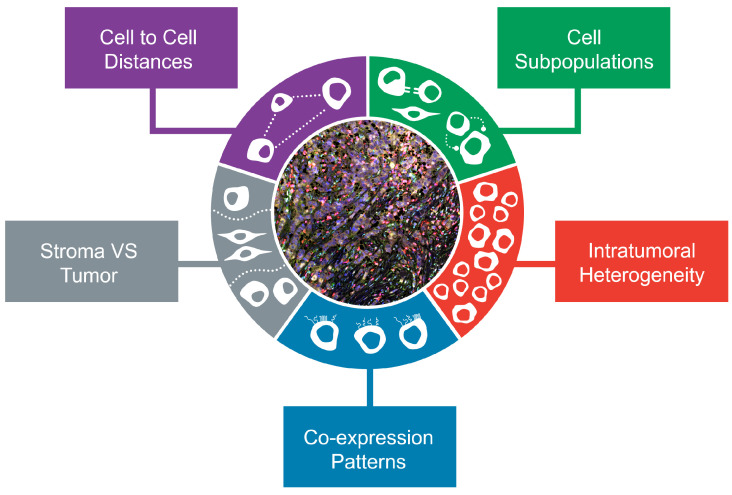
Hallmarks of multiplexed in situ TIME profiling. Different aspects which can be covered by the various multiplex assays and techniques enable a comprehensive characterization of the tumor microenvironment (TIME) in breast cancer.

In summary, novel methods have expanded the toolkit of translational cancer research applications and workflow by providing spatial information and multiplexing capacity in situ. The advantages and hallmarks of multiplexed profiling are depicted in Figure 1.

## 3. Applications of Multiplex Methods in Breast Cancer: TIME Composition, Spatiotemporal Heterogeneity and Prognostic Implications

In the past years, a great interest and intense efforts have been marked for the integration of the aforementioned emerging methods in breast cancer research. Several studies have adapted the advantages of such techniques in order to better characterize the TIME and address important questions in both the early and metastatic setting of the disease. We discuss hereunder how spatial methods and their aspects have been used to dissect the composition of TIME in terms of subpopulations and densities as well as in spatiotemporal heterogeneity in BC patient samples.

### 3.1. Overview of Multiplex In Situ Evaluation of Immune Response Heterogeneity in BC

Multiplex in situ assays allow for a more comprehensive characterization of BC TIME composition. On one hand, they can detect different subpopulations by various approaches. At first, lineage specific markers can be used such as T cell (CD3+, CD4+, or CD8+), B-cell (CD20+), NK-cell (CD56+), myeloid (MPO+, CD11c+), endothelial (CD31+) or fibroblastic (FAP+, SMA+, vimentin+), among others. Intriguingly, despite the fact that T cells comprise the most abundant immune population in BC TIME, TILs rates by mIHC/IF seem to modestly to strongly correlate with H&E TILs findings, in part due to panel selection and the scoring algorithm [60,61,62,63,64,65]. Furthermore, combinations of different markers have been used as surrogates for function, and include proliferative cells (CD3+Ki67+ [60], CD20+ki67+ [66]), tissue resident memory effector T cells (CD8+CD103+) [67], cytotoxic T cells (GZMB+CD8+) [66], regulatory T cells (CD4+FoxP3+) [68] and follicular helper T cells (CD4+CXCL13+CXCR5-) [66]. Moreover, multiplexing allows for simultaneous interrogation of several immune checkpoints such as PD1, TIM3 and LAG3, within their respective cell of origin [69,70,71]. On the other hand, all the aforementioned subpopulations are evaluated in situ within their niche, to better characterize the spatial organization and intratumoral heterogeneity of anti-BC immune response. Incorporation of different regions of interest (ROIs) in the analysis, tissue segmentation into tumoral and stromal compartments as well as distance analysis can reveal different localization patterns and correlations/co-existences of different subpopulations within BC TIME. Utilizing multiplexing across longitudinal analysis, either under the pressure of neoadjuvant treatment, or during metastatic dissemination, can further capture the temporal heterogeneity of anti-tumor immune response. In the following paragraphs we summarize the different aspects of spatial analysis that has been made so far in BC with divergent multiplex in situ methods and computational algorithms. An overview of the different studies and their characteristics is provided in Table 3.

### 3.2. Spatial Heterogeneity of Breast Cancer TIME

#### 3.2.1. Intratumoral Spatial Heterogeneity: Regions of Interest, Tissue Compartments, Cell-to-Cell Distances

Immune cell infiltration in TIME exhibits spatial heterogeneity [24]; thus, examination of small or limited number of regions of interest (ROIs) can potentially lead to sampling bias. Intriguingly, intra-section heterogeneity contributes more to differences in density of immune cells compared to inter-biopsy heterogeneity [72]. This is more apparent for B cells, as 75% of density differences is attributed to ROI examination within a slide. Besides this, B cells seem to be distributed in TIME independently of their stromal density [73].

Studying the distribution of immune subpopulations in tumoral and stromal compartments may overcome a weakness of the current evaluating systems. In concordance with H&E sTILs, the vast majority of immune markers is enriched in stromal compartments of BC [74,75,76]. Indeed, T cells (CD3+, CD4+ or CD8+), B cells (CD20+) and macrophages (CD68+) are commonly found in stroma [62,63,73,77]. However, tumoral compartments seem to be more infiltrated by T cells (CD3+, CD8+) and NK cells (CD56+) [62,73,77,78].

Intriguingly, immunophenotypes correlating with T cell activation (CD8+CD103+ memory effector, CD3+Ki67+ proliferating and GZB+CD8+cytotoxic) tend to be located intratumorally, or in close proximity to cancer cells [60,66,67]. In addition, tumoral HLA-DR expression, driven by IFNγ, correlates closely with stromal CD4+ density [74].

Description of cell-to-cell distances, interactions and neighbourhoods’ formation, may be useful for interrogating anti-tumor immune response spatial organization. Spatial analysis has demonstrated that presence of T cells (CD4+, CD8+) and B cells (CD20+) correlates with one another [62,72]. This correlation is highlighted by the presence of lymphocytic aggregates, consisting of purely T cells or tertiary lymphocytic structures (TLS) [66,72,73,79]. Besides this, B cells are known to be enriched in TLS, the presence of which can easily be missed [23,80]. Recently, with mIF/IHC TLS in BC have been described, characterized by close proximity of follicular helper T cells (CD4+PD1+) and proliferative B cells (CD20+Ki67+) within follicle-like structures. Functional activity was determined by a score generated from the combined expression of CD4+PD1+ Tfh, CD20+ki67+ B cells, and GZMB+CD8+ cytotoxic T cells [66]. Furthermore, immune infiltration seems also to correlate closely with other stromal cells of TIME. Immune cells are spatially proximal to endothelial cells (CD31+) [28], while CD25+ T cells have been found interacting with FAP+PDL2+OX40L+ CAFs [81].

In summary, multiple ROIs assessment, tissue segmentation and distance analysis are needed to address intra-tumoral heterogeneity of TILs (Table 3). Downsizing the analysis to a mere report of an average density across all ROIs, although easier, is an oversimplified approach.

#### 3.2.2. Spatial Heterogeneity of Immune Regulatory Proteins: The PD-L1 Paradigm

Several acellular factors are found within TIME, including positive (e.g., co-stimulatory molecules) and negative regulators (e.g., immune checkpoints) of immune response [25]. Among other molecules, PD-L1 is perhaps the most studied one with multiplex in situ methods. PD-L1 can be expressed by a variety of cell types in TIME and its expression is often associated with presence of TILs and other immune regulators [25,82]. Multiplexing techniques can be used to simultaneously evaluate PD-L1 within its cell of origin, in combination with other immune checkpoints and to interrogate its spatial heterogeneity [70].

**Table 3:** Breast cancer studies; Characteristics, Prognosis/Prediction, Heterogeneity.

Author	Journal	Year	Tissue Type	Disease Setting	Treatment	BC Type	Pts No	Assay	Panel	Scanning	Software	ROIs Number, Area (per Patient)	Tissue Segmentation	Prognosis/ Prediction	Spatial Heterogeneity
**Fluorescent Multiplex Immunohistochemistry**															
Angelis [62]	CCR	2019	WTS	Neo-adjuvant	Lapatinib trastuzumab	HER2+ BC	29	mIF	CD4, CD8, CD20, FoxP3, CD68, CK, DAPI	Vectra	InForm	5, 2 mm${}^{2}$	(CK+/DAPI+) (CK-/DAPI+)	Higher Stromal CD4, Tumoral CD4,CD20: Higher pCR	CD8 enriched In tumoral, CD4, CD8, CD20 Positive correlation
Brown [80]	CCR	2014	WTS	Neo-adjuvant	Taxane, anthracycline trastuzumab	all	87	mIF	CD3, CD8, CD20, CK, DAPI	PM-2000 hardware (HistoRx)	AQUA	At least 3 CK enriched	(CD3+/DAPI+) (CD3-/DAPI-)	Higher stromal CD4, CD8, CD20: Higher pCR	TNBC Higher CD20, CD8 densities
Graeser [61]	JIC	2020	WTS	Neo-adjuvant	Paclitaxel, gemcitabine, carboplatin	TNBC	66	mIF	CD4, CD8, CD73, PD1, PD-L1, CK7, DAPI	Vectra/Polaris	InForm	Whole slide	(CK7+/DAPI+) (CK7-/DAPI+)	On treatment CD4+PD1+: Higher pCR	NA
Griguolo [60]	Npj precision oncology	2021	WTS	Neo-adjuvant	Lapatinib, trastuzumab	HER2+ BC	65	MCISSS	CD3, CD4, CD8 Foxp3, Ki67, panCK, hematoxylin	NanoZoomer 2.0HT (Hamamatsu Photonics, Japan)	VISIOPHARM	Whole slide	(CK+/HTX+) (CK-/HTX-)	On treatment TumoralCD8+ higher pCR	Ki67+CD3+ Higher densities closer to cancer cells, enriched in HR-
Kearney [63]	SABCS	2021	WTS	Neo-adjuvant	Anthracycline Trastuzumab pertuzumab	HR+/ HER2+ BC	28	mIF	CD3, CD8, CD68, FOXP3, Pan-CK, DAPI	NA	HALO	NA	CK+ CK-	Higher CD3+CD8-FoxP3- higher pCR	NA
Yam [71]	CCR	2021	WTS	Neo-adjuvant	doxorubicin, cyclo-phosphamide, paclitaxel	TNBC	102	mIF	PDL1, PD1, CD3, CD68, Pan-CK, DAPI	Vectra 3.0	InForm	NA	CK+ CK-	Higher CD3+/CD68+ ratio, CD3-cancer proximity higher pCR	PD-L1 expression: tumor (more common) and stromal compartments, CD3+ PD1+ rare population
Janiszewska [83]	JCIinsight	2021	WTS	Neo-adjuvant	Trastuzumab emtansine, pertuzumab	HER2+ BC	20	mIF	20	NA	NA	NA	CK+ CK-	pCR: higher CD8, RD:GZM+ mf closer to cancer, immune cells less proximal to vimentin+ cells	NA
Egelston [67]	JCIinsight	2019	WTS	Adjuvant	doxorubicin, cyclophospamide hamide, paclitaxel	TNBC	25	mIF	CD8, CD103, CD69, pan-CK, DAPI	Vectra 3.0	InForm	multiple	(CK+/DAPI+) (CK-/DAPI+)	Higher Tumoral CD8+CD103+: Better RFS	CD8+CD103+ Enriched closer to cancer cells
Millar [84]	Cancers	2020	TMA	Adjuvant	CMF, anthracycline	all	485	mIF	CD3, CD8, CD20, CD68, Fox P3, PD-1, PD-L1, PanCK, DAPI	Vectra/Polaris	InForm	1, 780 μm${}^{2}$	(CK+/DAPI+) (CK-/DAPI+)	Combined Stromal lower CD8, CD20: Shorter OS	NA
Garaud [79]	JCIinsight	2019	WTS	Adjuvant	Chemotherapy	HER2+ BC, TNBC	249	mIF	CD4, CD8, CD20, FoxP3, CD68, CK, DAPI	Vectra/Polaris	InForm	NA	NA	Higher CD20: Favorable prognosis	Description of Lymphocytic aggregates
Costa [81]	Cancer Cell	2018	NA	NA	NA	TNBC	NA	mIF	CD25, FAP, PDL2, OX40L, DAPI	HistoFluor microscope	manual	NA	NA	NA	FAP+PDL2+ OX40L+: T cell exhaustion
Wortman [73]	Npj Breast Cancer	2021	WTS	Adjuvant	Chemotherapy	TNBC	36	mIF	CD3, CD4, CD8, FOXP3, CD20, DAPI, PanCK	Vectra 3.0	InForm	NA	NA	Higher tumoral Distribution CD3,CD20: Better RFS	CD3, CD20: Description of Aggregates
Mani [72]	Breast cancer research	2016	WTS	NA	Surgery	NA	31	mIF	CK, DAPI, CD3, CD8, CD20	NA	AQUA	6–50	NA	NA	Approximately 70% of spatial Heterogeneity of B cells: Within same tissue section
O’Meara [85]	SABCS	2021	NA	NA	NA	all	132	mIF	CD8, FoxP3, PD1, PDL1, CK	Vectra	NA	NA	NA	NA	HR+ Immune mark.: Correlates with Grade, Recurrence score
Shimada [86]	SABCS	2021	NA	NA	NA	HR+ BC	5	mIF	9-21 proteins	CyteFinder microscope	MCMICRO	NA	Tumor Stroma Immune cells	NA	Description Of cold, hot Excluded TIME
Noel [66]	JCI	2021	WTS	Adjuvant	Chemotherapy, HER2 targeted	HER2+ BC, TNBC	48	mIF	CD4, CD20, PD-1, ICOS, Ki-67, Foxp3	Vectra/Polaris	NA	NA	NA	Active TLS: Favorable prognosis	Active TLS: Combined CD4+PD1+, CD20+ki67+, GZMB+CD8+
Bedard [68]	SABCS	2021	NA	Metastatic	INT230-6 (cisplatin and vinblastine) pembrolizumab	TNBC	3	mIF	CD4, CD8, FoxP3	NA	NA	NA	NA	NA	Metastasis Lower CD4,CD8 Higher FoxP3
Zhu [87]	J.Immun. Cancer	2019	WTS	Metastatic	NA	all	5	mIF	CD8, FoxP3, CD68, CD20, CK, DAPI	NA	NA	NA	NA	NA	Metastasis Higher CD68
He [69]	Plos One	2020	WTS	Metastatic	Chemotherapy	TNBC	10	mIF	CD4, CD8, FOXP3, CD20, CD33, PD1, CK, DAPI	Vectra	InForm	NA	CK+ CK-	Higher PD1+CD8+ PD1+CD4+ Better outcome	Metastasis Lower Str. CD8+, PD1+CD8+
**Ultivue InSituPlex**															
Ahmed [70]	CCR	2020	WTS	Neo-adjuvant	nab-paclitaxel, doxorubicin, cyclophosphamide, durvalumab	TNBC	45	InSitu Plex	CD8,CD68,PDL1, CK, Sox10, Hoechst	PM2000 microscope (HistoRx)	AQUA	NA	CK+, CK- CD68-, CK- CD68+	pCR vs RD: higher baseline PDL1 expression and similar CD68 densities	PD-L1 expression: on both stromal and tumoral compartments
**GeoMx Digital Spatial Profiling**															
Carter [75]	SABCS	2020	WTS	Neo-adjuvant	taxane	HR+/ HER2- BC	39	DSP	58 proteins	GeoMx	NA	6, 600 μm${}^{2}$	(CK+/SYTO13+) (CK-/SYTO13+)	After NAT: LAG3 expression: Residual disease	Most immune Markers: Stromal enrichment
McNamara [77]	Nature Cancer	2018	WTS	Neo-adjuvant	Trastuzumab lapatinib	HER2+ BC	28	DSP	40 proteins	GeoMx	NA	4, 464–666 μm${}^{2}$	CK+ CK-	On treatment Expression CD45, CD56 Higher pCR	Compartment enrichment Tumoral CD56, B7H4 PDL1, IDO Stromal CD3, CD4, CD8, CD68
Carter [76]	SABCS	2020	TMA	adjuvant	Chemotherapy	TNBC	167	DSP	58 proteins	GeoMx	NA	1, 600 μm${}^{2}$	(CK+/SYTO13+) (CK-/SYTO13+)	Stromal LAG3 Higher PFS	Compartment Enrichment Tumoral CD27, HLADR, IDO1 PDL1 Stromal PDL1
Stewart [74]	Scientific reports	2020	WTS	Adjuvant	Chemotherapy	TNBC	10	DSP	39 proteins	GeoMx	NA	6, 300 μm${}^{2}$	CK+ CK-	No relapse: Tumoral HLA-DR, IDO1, B2M Stromal CD45, CD4, PD-1	Most immune Markers Stromal
Kulasinghe [88]	Frontiers In Oncology	2022	TMA	Adjuvant	Chemotherapy	TNBC	24	DSP	68 proteins	GeoMx	NA	1, NA	CK+ CK-	Responders Tumoral higher GZMA, STING, Fibronectin Lower CD80 Stromal Lower 4-1BB	NA
Carter [89]	CCR	2021	TMA	NA	Chemotherapy untreated	TNBC	184	DSP	58 proteins	GeoMx	GeoMx software	1, 600 μm${}^{2}$	CK+, CK-/SYTO13+	NA	PD-L1+: immunologically hotter (mf, T cells, checkpoints (IDO-1), antigen presentation, STING)
Leon-Ferre [90]	SABCS	2021	TMA	NA	Surgery	TNBC	111	DSP	58 proteins	GeoMx	NA	1, NA	CK+ CK-	NA	LAR vs non-LAR TNBC Lower Stromal CD45, CD14, IDO1 Tumoral CD45, B7H3
Schlam [78]	JTM	2021	WTS	Metastatic	Chemotherapy HER2 targeted	HER2+ BC	8	DSP	70 proteins	GeoMx	NA	2	CK+ CK-	NA	Metastasis Lower CD3, CD8 Tim-3, CD27, 4-1BB
**Mass Spectrometry Immunohistochemistry**															
Bianchini [91]	SABCS	2021	WTS	Neo-adjuvant	atezolizumab, carboplatin, nab-paclitaxel	TNBC	243	IMC	43 proteins	TOF	NA	NA	NA	Higher pCR: density APC PDL1+IDO+, spatial correlation of CK+ and CD8+PD1+, CD8+GZMB+, CD20+	NA
Keren [28]	Cell	2018	WTS	NA	Surgery	TNBC	41	MIBI- TOF	36 proteins	TOF	NA	1, 800 μm${}^{2}$	NA	Compartmentalized pattern longer OS	Hot tumors Were divided Into “Mixed”, “Compartmentalized”

Abbreviations: APC: antigen presenting cell, BC: breast cancer, RD: residual disease, CK: cytokeratin, DSP: digital spatial profiling, HR: hormone receptor, IMC: imaging mass cytometry, MIBI-TOF: multiplex ion beam imaging with time of flight, mIF: multiplex immunofluorescence, MCISSS: Multiplexed Immunohistochemical Consecutive Staining on Single Slide, OS: overall survival, pCR: pathologic complete response, PFS: progression-free survival, RFS: relapse-free survival, TLS: tertiary lymphoid structure, TMA: tissue microarray, TNBC: triple negative breast cancer, TIME: Tumor immune microenvironment, WTS: whole tissue section.

Similarly for TILs, multiple ROIs are required to capture PD-L1 intra-tumoral heterogeneity [72]. In BC, PD-L1 expression is seen on cancer cells, T cells (CD3+, CD4+, or CD8+) and myeloid cells (CD68+, CD163+ macrophages, CD11+ myeloid derived suppressor cells) [70,71,92]. Tissue segmentation reveals different expression patterns between tumoral and stromal compartments. In CK enriched compartments, PD-L1 is mostly expressed by cancer cells or T cells, while in stromal compartments expression is mediated by macrophages or T cells. In addition, cancer-immune mixing level has a further impact on PD-1/PD-L1 axis.

In “mixed” tumors, PD-L1 and PD-1 expression are noted in cancer cells and CD8+ T cells, respectively. In contrast, “compartmentalized” tumors express PD-L1 on CD11+ myeloid derived suppressor cells and PD-1 on CD4+ T cells [28]. Regarding other immune regulators, PD-L1 expression in TNBC is associated with both negative (PD1, Lag3, IDO1 and FoxP3) and positive (HLA-DR and CD40) regulators of immune response [28,89]. Tissue segmentation shows even distribution of PD-L1 and other checkpoints between stromal and tumoral compartments [76,77]. However, tumoral compartments seem to exhibit stronger expression of negative regulator IDO and positive regulators CD127, CD27, B7H4 [75,76]. Recently, in a large cohort of 184 untreated TNBC cases, PDL1 positivity was associated with “hotter” TIME compared with PDL1- compartments [89]. Taken together, multiplex methods can be applied to comprehensively study the expression of immune-related molecules and such efforts could be of help to re-evaluate the role of PD-L1 as predictive biomarker to ICI.

#### 3.2.3. Inter-Patient Spatial Heterogeneity: Characterization of Spatial Organization of BC TIME

Inter-patient heterogeneity could also be demonstrated with spatial multiplexing, especially within the various BC subtypes. For HER2+, BC proliferative Ki67+CD3+ T cells densities were found to be higher for HR- compared to HR+ disease [60]. TNBC exhibits the higher TILs rates among BC types, and this has been also observed using mIF/IHC with CD20+, CD8+, CD8+PD1+, FoxP3+ and PDL1+ densities to be higher in this subtype [80,85]. Moreover, Leon-Ferre et al. used DSP and demonstrated that LAR TNBC is immunologically “cold” compared with non-LAR TNBC [90]. Recently, Cy-TOF method was used to comprehensively classify TIME of BC at single cell level [93,94]. Jackson et al. concluded that TNBC harbor two types of TIME, either one heavily infiltrated by cancer cells with only scarce stroma, or another associated with T cell or macrophage rich infiltration [93]. HR+ tumors are generally infiltrated by a lower number of immune cells, compared with HER2+ and TNBC. However, a subset of HR+ BC contained higher densities of immune cells, located in some of the ROIs examined [93]. In another study, immune infiltration and PDL1 expression positively correlated with pathologic grade and Recurrence Score [85].

Interactions of TIME with other cell types such as CAFs has also been elucidated. Luminal BC seems to be mainly enriched in CAFs and divergent CAFs types have been described with heterogeneous immunophenotypes, including vimentin+, SMA+ or CD68+ [93,94], with luminal B tumors being infiltrated more from CD68+ CAFs compared to Luminal A which are infiltrated from SMA+ CAFs [94].

Multiplex in situ methods can be also used to classify tumors as “cold”, “excluded” or “hot”, based on tumor-immune interaction patterns of immune infiltration. In a landmark paper, Keren et al. demonstrated that immune cell infiltration of immunologically “hot” TNBC tumors can be subdivided into “mixed” and “compartmentalized” [28]. “Mixed” tumors have immune cells mixed with carcinoma cells, while in “compartmentalized” immune cells are spatially distinct from neoplastic cells. In tumor-immune border of “compartmentalized” tumors, cancer cells express HLA-DR, B cells are depleted, while granulocytes (MPO+) are enriched [28]. Intriguingly, spatial distribution of immune cells can also classify hormone receptor-positive BC (HR+ BC), into “cold”, “restricted” or “hot” [86].

Overall, these studies show that high-multiplexing level allows for new classifications of BC TIME (Table 3), complementary to those introduced by gene expression profiling [29,95,96]. In this way, the biologic relevance of rare subpopulations as well as novel histological patterns of immune response can be described.

### 3.3. Temporal Heterogeneity of Breast Cancer TIME

#### 3.3.1. Longitudinal Evaluation under Treatment Pressure at the Neoadjuvant BC Setting

A dynamic, longitudinal evaluation of inflammatory changes within BC TIME is of utmost importance to better understand anti-tumor immune response. Some studies have used mIF/IHC and DSP to longitudinally evaluate immune response under the pressure of neoadjuvant therapy [60,61,75,77]. Treatment with either chemotherapy or HER2 targeted therapies lead to increase of markers associated with cellular immunity, except for a study [75], which demonstrated that chemotherapy globally downregulated the expression of immune markers. Compared with baseline, increased infiltration of CD8+ [60,77], FOXP3+ [60], CD8+PD1+ [61] and PD-L1+ [61] was noted, while CD3+KI67+ [60] cells decreased. Interestingly, intra-tumoral spatial distribution of T cells also changed, with CD3+ and CD8+ T cells being recruited closer to cancer cells, either in tumoral [60,61,77] or proximal stromal compartments [60]. In summary, these studies demonstrate that multiplex methods can be used to evaluate the dynamic changes of TIME, under the pressure of neoadjuvant therapy.

#### 3.3.2. Intra-Patient Heterogeneity: From Primary Breast Cancer to Metastatic Progression

Multiplex methods can also be employed to characterize TIME composition changes from primary site to metastasis. Metastatic tumor areas have been generally considered as immunologically “colder” compared with primary disease [78], with low TIL counts at metastatic tissue [13] and highly discordant PD-L1 expression compared to primary tumors [97,98]. In two previous reports, a decrease of T cells (CD3+, CD8+) density in both stromal [69,78] and tumoral compartments [78] was noted, while macrophages seemed to be increased [87]. Furthermore, functional status of TILs was also affected. In another study, lower infiltration of dysfunctional (PD1+) T cells was noted and linked to worse outcomes [87]. Furthermore, a DSP analysis of HER2+ BC showed decreased expression of checkpoint Tim-3 and co-stimulatory CD27, and 4-1BB molecules [78]. However, even for advanced disease, combinational treatment of chemotherapy and immunotherapy was shown to increase CD4+ and CD8+ counts compared to FoxP3+ cells [68]. In summary, these studies show that dynamic assessment of immune response in the context of temporal heterogeneity is feasible and informative with multiplex methods (Table 3).

### 3.4. Prognostic and Predictive Implications of Multiplexed Spatial Profiling in Breast Cancer

The prognostic role of immune infiltrate in BC has been explored during the last decade mostly via H&E assessment. It is well established, that higher rates of baseline H&E sTILs confers favorable outcomes in chemotherapy treated, early-stage HER2+ BC and TNBC, while the same does not apply for HR+/HER2- disease [15]. Regarding clinical significance of immune subpopulations, current knowledge remains limited. In a pooled analysis of 12439 BC patients, presence of intratumoral or stromal CD8+ T cells, evaluated by IHC, predicted reduced BC-related mortality in both HR+/HER2+ and HR- settings [26]. Multiplex in situ methods may cover the gap of clinical significance between quantity and quality of immune infiltration. Digital enumeration and spatial distribution analyses, generate divergent prognostic metrics, which can be grouped into lineage-, spatial- and functional-specific assessments (Table 3). Of note, clinical focus should not be placed solely on T cells, as studies have shown that B cell evaluation carries prognostic information [66,79,80]. Similar to H&E sTILs, combined scores of stromal CD4+, CD8+, CD20+ correlated with better outcome in BC [62,80]. In luminal BC, patients with concurrent high levels of CD8+ and CD20+ cells had significantly increased overall survival [84]. In addition, expression of LAG3 correlated with residual disease after chemotherapy [76]. Regarding HER2+ BC, higher levels of on-treatment T cells (CD8+) [60] and NK cells (CD56+) [77], correlated with increased pCR with HER2-targeted therapy. Interestingly, in HR+/HER2+ BC, spatial proximity of CD8+ T cells and helper T cells (CD3+CD8-FoxP3-) predicted pCR [63]. Furthermore, in a small cohort of trastuzumab emtansine treated patients, close proximity between GZM+ macrophages and cancer cells was associated with residual disease [83]. In TNBC, prospective retrospective analysis of 102 cases treated with standard neoadjuvant chemotherapy, showed that higher CD3+/CD68+ ratio and CD3+ proximity to cancer cells independently predicted pCR [71]. However, in other settings, spatial analysis has produced discordant results due to limited sample size and use of different analysis pipeline [28,73]. On one hand, high spatial dispersion of CD3+ and CD20+ correlated with better RFS [73]. On the other hand, organized “compartmentalized” histology, predicted better overall survival compared with a “mixed” pattern [28]. Furthermore, the identification of TLS in BC TIME may be of clinical importance, as, in various solid cancer types, TLS presence has been associated with better outcomes [99,100,101]. Intriguingly, the functional status, and not the number of TLS, seems to correlate with DFS [66]. Evaluation of functional status of immune cells in TNBC revealed that increased density of tumoral CD8+CD103+ memory T cells correlated with better RFS [67]. Similarly, concurrent expression of tumoral HLA-DR, B2M, IDO-1, STING and GZMA as well as stromal PD-1 characterized non-recurrent TNBC tumors [74,88]. In addition, higher tumoral and stromal PD-L1 expression has been associated with response to chemoimmunotherapy combination [70]. Moreover, baseline stromal LAG3 expression and altered densities of CD4+PD1+ T cells after three weeks of chemotherapy were favorable prognostic factors [61,76]. To date, the largest application of imaging mass cytometry has been applied in 243 TNBC of NeoTRIPaPDL1 phase III trial, where high densities of PDL1+IDO+ antigen presenting cells and spatial correlation of cancer cells with T cell subsets (CD8+PD1+, CD8+GZMB+) and B cells (CD20+) accounted for higher pCR rates in atezolizumab arm, independent of sTILs and PD-L1 IHC [91]. In summary, multiplex in situ evaluation of immune cells’ densities, spatial distribution and functional status may provide the necessary step forward in identification of TIME-related biomarkers (Table 3). Of note, few of these studies have tested locked-down classifiers against pre-specified hypotheses. This is a major caveat against the conclusions drawn and independent validation is needed.

### 3.5. AI-Assisted Evaluation of TIME on H&E Breast Cancer Tissue Images

In the past years, new methods based on image analysis of H&E stained images have been developed for the assessment of TIME and TILs so that the setbacks of morphology-based TILs evaluation could be also overcome. Such methods are based mostly on deep- and machine-learning algorithms coupled with artificial intelligence approaches [102]. Recently, recommendations have been published from the international immuno-oncology group to aid towards clinical validation [103]. In BC, the clinical significance of digital TILs enumeration has been explored [65,104,105,106,107]. In a recent study by Bai et al., a semi-supervised neural network was developed for the scoring of digital TILs in 920 early TNBC patients and demonstrated that increasing digital TILs density was a favorable prognostic factor [104]. Furthermore, retrospective analyses of prospective cohorts that recruited a large sample of BC patients, concluded that digital TILs counts independently predicted pCR [105,106]. The favorable prognostic significance of digital TILs was also validated in a cohort of 146 BC, especially for HER2+ BC and TNBC [107]. All the aforementioned studies including large number of patients, suggest that digital TILs densities could be of clinical importance. However, since H&E manual TILs scoring remains the “gold standard”, the concordance of digital and manual evaluation needs to be further assessed. Indeed, significant correlations between the algorithm’s TILs scores and the pathologist’s read sTILs assessments have been reported [64,104]. Intriguingly, multiplex methods can also digitally assess lymphocytic subpopulations densities and the interplay between TILs and CAFs in H&E-stained images has been also studied with the use of digital pathology. A recent study evaluated the spatial relations of CAFs and TILs within TIME of HR+ BC. Using AI-based analysis on H&E slides, the cellular densities and spatial proximity of TILs and CAFs were calculated. Hierarchical clustering revealed five TIME types, with one type/cluster being associated with IFNγ gene expression, high TILs and CAFs densities mixing with each other. Of note, mixing histology was a favorable prognostic factor, independently of other clinicopathologic factors, TILs and CAFs densities [108]. Overall, digital evaluation of TILs, in terms of enumeration and spatial distribution is likely to become an invaluable tool for cancer research. Incorporation of immune subpopulations in the analysis of multiplexed data will expand further TIME understanding.

## 4. Discussion

Over the past few years, immunotherapy has generated promising results in the treatment of patients with TNBC. However, the mechanisms that govern response and resistance to checkpoint inhibition are poorly understood. A deeper understanding of the underlying complex biology of the TIME may pave the way for the discovery of novel immune-related biomarkers. Multiplex in situ methods seem to be powerful tools towards this effort, overpowering or complementing other TIME evaluation techniques, especially when only small tissue specimens are available for analysis [16]. On one hand, they can identify patterns not detected by conventional H&E or IHC histopathological techniques. They enable the simultaneous detection of densities, subpopulations and functional states of immune cells as well as different immune stimulators or suppressors within their cellular context. On the other hand, they can effectively capture spatial heterogeneity of anti-tumor immune response, complementing on sequencing-based methods. Gene expression signatures and single cell-omics are devoid of spatial information [109]. In bulk-omics, the signal is dominated by the predominant cellular population and rare, yet important, populations are missed. Although single cell-omics can identify rare populations, their biologic relevance is not fully characterized as their spatial interrelations are lost [109].

Protein-based multiplex methods remain of utmost importance, since protein expression is a direct estimation of functionality. However, RNA-based methods (spatial transcriptomics) have also been established and complement protein-based studies. To date, two types of spatial transcriptomic approaches exist and they have been extensively reviewed in elsewhere [110,111]. At first, sequencing approaches include: laser capture microdissection, mRNA capture, microfluidics (DIBIT-sequencing), in situ sequencing, fluorescence in situ sequencing (FISSEQ) and GeoMx DSP, which could also be employed to assess mRNA expression in situ, apart from protein evaluation. Secondly, fluorescent in situ hybridization (FISH) approaches include: single-molecule FISH (smFISH), Spectral barcoding, Spatial barcoding, osmFISH, multiplexed error-robust fluorescent in situ hybridization (MERFISH), sequential FISH (seqFISH, seqFISH+) and RNAscope. To our knowledge, limited studies have used such approaches to evaluate breast cancer TIME heterogeneity [112].

Overall, multiplex in situ assays represent an invaluable tool for dissecting breast cancer TIME at a spatial resolution, going beyond H&E TILs and PDL1 IHC. The combination of longitudinal and spatial analysis at ROI, tissue compartment and cell-to-cell interaction level, allows for in situ evaluation of divergent, and even rare, immune subpopulations as well as immune checkpoints within their cell of origin. This paves the way for spatial interrogation anti-tumor immune response organization, hypothesis generation regarding immune cells’ function as well as identification of novel immune related biomarkers. Furthermore, novel taxonomies of BC TIME emerge and allow for improved identification of BC patients with “inflamed”, “excluded” and “cold” breast tumors [28,93,94].

Before introducing multiplex in situ methods into clinical practice, several analytic challenges need to be overcome. It was recently demonstrated that different methods yield discrepancies for immune infiltrate assessment [113]. This observation is of utmost importance, since previously non-reproducible results have compromised the potential clinical significance of explored biomarkers [7,8,18]. In this direction, efforts have been undertaken to standardize recommendations for mIHC/IF [38,114] and DSP [115] but several questions still need to be addressed.

Firstly, the optimal number of markers within a panel is not known. Multiplex IHC/IF approaches use narrow, easier to validate panels, which makes them more relevant in the context of a clinical trial. In addition, cost, scanning times and image analysis labor are significantly lower compared with CODEX, or MS-IHC based approaches. However, narrowing down analysis to a limited number of biomarkers studied can potentially lead to an oversimplification of the underlying biology. Smaller antibody panels may overlook specific subpopulations and may be unable to characterize the whole spectrum of immune cell dysfunction or the interplay between different immune regulators [116]. In contrast, high-plex methods may provide a more global characterization of TIME; however, increased complexity generates difficulties regarding panel validation, intensity profile, cell segmentation and phenotyping [39,117]. In addition, the sensitivity of MS-IHC approaches compared with single IHC is yet to be determined [38].

Secondly, the optimal number and selection method of regions of interest needs to be determined. Ideally and in order to effectively tackle spatial heterogeneity, examination of whole tissue section is required, but this significantly increases both cost and image analysis labor. As multiplexing level increases, the available ROI is often limited to less than 1 mm^2^. Multiplex IF/IHC allows for evaluation of larger (even whole slide) ROIs, while mass cytometry-based approaches are limited to one small (800 μm${}^{2}$) ROI. Selecting ROIs generates another challenge, as manual selection is arbitrary and is susceptible to selection bias. On the other hand, image curation by a pathologist can exclude artifacts, fibrotic or necrotic areas that can potentially confound image analysis results. Considering that spatial heterogeneity is the cardinal factor of interobserver variabilities during H&E TILs evaluation, ROI selection is a critical step for quality research. In order to circumvent this issue, it is generally recommended to evaluate as many ROIs as possible [38,65]. Berry et al. addressed this question by comparing random with hot-spot, deep-learning sampling and demonstrated that hot-spot sampling was not inferior in terms of predicting immunotherapy benefit [27]. Thirdly, regarding the image analysis workflow, guidelines for standardization are an unmet need. Several technological limitations exist for scanning speed, image generation, resolution level, measurements, and software performance. Furthermore, several challenges exist across the pipeline of multiplex (multispectral) images analysis. The first challenge regarding multiplex data and whole slide imaging in general is related to the acquisition and curation of such data. Due to their large size, storing, retrieving and processing such images requires careful planning and optimization of the storage infrastructure [27,110]. Another challenge concerns the analysis of the multiplex images (e.g., cell phenotyping), including cell segmentation and classification. Incorrect segmentation of cells, either by splitting individual cells or by merging multiple ones can result in over/under-estimation of specific types of cells [27]. Balanced representation of cell populations within the training and testing sets is of paramount importance, since training on unbalanced datasets can lead to model overfitting to the over-represented class, while validating on such datasets produces unreliable performance results. Cellular segmentation can be laborious and challenging, especially when cells with different shapes and sizes are mixed [118,119,120]. The level of complexity increases further when multiple biomarkers need to be assessed, as cell borders can be poorly defined [49]. To overcome such challenges, new approaches are being developed to assess biomarker expression at pixel-level instead of cellular-level [49]. Moreover, results from proximity studies between cancer cells and immune cells may be affected by difference in cell densities. Towards this end, performing proximity analysis between compartments with similar levels of cellular infiltration has been suggested [121]. Other challenges could be posed within the image analysis pipeline, such as the development of good-quality data, existence of inter-center and inter-vendor variability, as well as intra- and inter-observer variability either during tissue sample preparation or during image acquisition. Controlling these analytical checkpoints is a significant factor for the development, validation and interpretation of robust and accurate models and image analysis tools. To ensure reproducibility, an extensive and detailed report of image analysis workflow is warranted. All the aforementioned analytical challenges are summarized in Table 4.

## 5. Concluding Remarks

Multiplexing methods are very likely to revolutionize our view of breast cancer TIME. All the aforementioned analyses have been implemented on breast cancer research for dissecting TIME at a spatial and multiplex resolution. Scarce data have been reported so far in a few descriptive studies, while others report on prognostic implications, sometimes with discordant results. From a clinical perspective, despite the promising initial data, prospective validation within adequately powered clinical trials is required. Longitudinal evaluation of tissue samples would be of high interest since inflammation and immune phenotypes are dynamic and change over time. Validation, optimization, standardization and detailed reporting is needed to achieve reproducible results. Therefore, the establishment of “multiplex/multi-omics tumor boards” including oncologists, pathologists, molecular biologists/immunologists, computational scientists and bioinformaticians could serve as a future perspective in order to better integrate and interpret multi-omics data/results in clinical benefit and practice.

## Figures and Tables

**Table 2:** Available software for multiplex analyses, compatible with the various platforms and assays.

Software (Company)	Analyses/Capacity
Inform [56] (Akoya Biosciences)	multispectral unmixing, tissue and cell segmentation, cellular phenotyping
MultiOmyx [37] (Neo Genomics)	quantitative analysis at the cell-level, spatial analytics
HALO [57] (Indica Labs)	immune cell population contexture analysis segmentation and classification
Visiopharm [58]	cellular identification spatial profiling
RSIPVision [59]	nuclei detection, segmentation and cellular colocalization
QuPath [40]	cell segmentation and classification

**Table 4:** Analytic challenges of multiplex in situ methods.

Multiplexing Level	
High	Challenging panel validation, increase in image analysis labor/difficult cell phenotyping
Panel Validation	
Section thickness	Affects staining intensity and tissue autofluorescence
Staining sequence of primary antibodies	Can deal with epitope instability and cross reaction of primary antibodies
Fluorophores, Co-localization in the same cellular compartment, Low abundance epitopes	Selection of spectrally separated fluorophores; selection of more intense fluorophores
Staining pattern	For each antibody, staining pattern in multiplex image should be identical to single-plex immunohistochemistry
Regions of interest	
Number	Whole tissue section resemblance: as many as possible evaluation
Prior selection	Potential selection bias
Statistical analysis	
Optimal statistical method	Hierarchical linear modeling: statistical power improvement over *t*-test.
Cut-off	Biomarkers expressed by various cell types: establishment of single positivity threshold is difficult
Image Analysis	
Storage	Extreme data sizes
Cell phenotyping	Difficult cellular segmentation: 1. Mixed cells with different shapes and sizes 2. High multiplexing level
Distance analysis	Densities can be confounding factor: Possible solution proximity analysis between areas with similar levels of cellular infiltration
Bias	Inter-center and inter-vendor variability, as well as intra- and inter-observer variability either during tissue sample preparation or during image acquisition.
